# Interaction between Neuromelanin and Alpha-Synuclein in Parkinson’s Disease

**DOI:** 10.3390/biom5021122

**Published:** 2015-06-05

**Authors:** Shengli Xu, Piu Chan

**Affiliations:** 1 Beijing Institute of Geriatrics, Xuanwu Hospital of Capital University of Medical Sciences, No.45 changchun St., Xicheng District, Beijing 100053, China; E-Mail: xushngli_xw@163.com; 2 Parkinson’s disease Center of Beijing Institute for Brain Disorders, Beijing 100053, China

**Keywords:** alpha-synuclein, Parkinson’s disease, neuromelanin, oxidative stress

## Abstract

Parkinson’s disease (PD) is a very common neurodegenerative disorder characterized by the accumulation of α-synuclein (α-syn) into Lewy body (LB) inclusions and the loss of neuronmelanin (NM) containing dopamine (DA) neurons in the substantia nigra (SN). Pathological α-syn and NM are two prominent hallmarks in this selective and progressive neurodegenerative disease. Pathological α-syn can induce dopaminergic neuron death by various mechanisms, such as inducing oxidative stress and inhibiting protein degradation systems. Therefore, to explore the factors that trigger α-syn to convert from a non-toxic protein to toxic one is a pivotal question to clarify the mechanisms of PD pathogenesis. Many triggers for pathological α-syn aggregation have been identified, including missense mutations in the α-syn gene, higher concentration, and posttranslational modifications of α-Syn. Recently, the role of NM in inducing α-syn expression and aggregation has been suggested as a mechanism for this pigment to modulate neuronal vulnerability in PD. NM may be responsible for PD and age-associated increase and aggregation in α-syn. Here, we reviewed our previous study and other recent findings in the area of interaction between NM and α-syn.

## 1. Pathological Characteristics and Aetiology of Parkinson’s Disease

Parkinson’s disease (PD) is the second most common neurodegenerative disorder. The clinical symptoms of PD include motor and non-motor symptoms. The main motor symptoms of PD are bradykinesia (resting tremor), rigidity, and postural instability [1,2,3]. It is believed that the progressive loss of neuromelanin (NM)-containing dopaminergic neurons in the substantia nigra pars compacta (SNpc) is the cause of motor symptoms in PD. Signaling between the SN and the striatum is involved in controlling muscle movements; therefore the resultant loss of nigro-striatal pathway signaling can explain the classical motor symptoms of PD [2,3]. “Dopamine (DA) replacement” pharmacotherapy is relatively effective at reducing the motor disturbances [4]. The non-motor symptoms include autonomic dysfunction (orthostatic hypotension, sphincter disturbances, and/or constipation), cognitive changes, psychiatric effects (depression, psychosis, and/or impulse control disorder), sensory symptoms (pain and/or aching), restlessness, and sleep disturbances [5,6]. These non-motor symptoms may result from disturbances of other neurotransmitter pathways, such as cholinergic, serotonergic, or GABA-ergic and they respond relatively poorly to dopaminergic therapies [7].

The aetiology of PD is often explained by the interaction between environmental and genetic factors. Environmental studies have identified significant risk factors for PD, such as the exposure to pesticides, herbicide, and metal irons [8]. Many of the recent studies reported that some rare genetic causes of PD relate to the mutations in genes. These genes include SNCA [9,10], the leucine-rich repeat kinase 2 (*LRRK2*) [11], glucocerebrosidase (*GBA*) [12], *Parkin* [13], *DJ-1* [14], *PINK1* [15] *HLA* [16] and *MAPT* [17] genes. In these genetics risk factors for PD, the most important is SNCA; the gene responsible for the expression of α-syn. To date, six PD-linked point mutations in *SNCA* have been identified, comprising the A30P [18], A53T [19], E46K [20], H50Q [21], G51D [22], and A53E [23].

On the other hand, aging is the biggest known risk factor for the development of idiopathic PD and it is crucial to understand its role. With advancing age, a number of processes essential for the function of SN neurons including DA metabolism, wild type mitochondrial DNA copy number and protein degradation decline [24]. A decline in wild type mtDNA copy number will lead to a decrease in ATP production and a reduction in efficient protein degradation will affect the function of neurons [25]. DA metabolism generates a significant amount of reactive oxygen species that will affect a number of different processes within the neurons [26]. In young and healthy SN neurons, toxic DA metabolic products could be formed in the dark pigment of NM. NM accumulates in double membrane autophagic vacuoles, preventing neurotoxic effects of free neuromelanin in cells exposed to this pigment [27]. However, in aging SN neurons, there is an age-related high content or overall accumulation of NM in the SN. The accumulation NM, which overload of toxic metals or compounds, can potentially result in increased oxidative stress and decreased transformation of toxic DA metabolic products into NM [28]. Moreover, the accumulation of NM may induce the expression and aggregation of α-syn, increasing toxic insults to NM containing SN neurons [29]. NM that leaks from degenerating neurons may contribute to the degeneration of DA neurons in PD by activating microglia [30]. Those toxic mechanisms cause the loss of vulnerable neurons, once this cell loss reaches a certain level, the symptoms of PD develop.

## 2. Parkinson’s Disease and α-Synuclein

### 2.1. α-Synuclein is Linked to the Pathogenesis of Parkinson’s Disease

The involvement of α-syn in PD was initially identified through genetic linkage studies in a small number of families [9], including mutations as well as gene duplications [31] and triplications [32]. Up to now, six missense mutations in α-syn gene have been linked to the pathogenesis of PD, comprising A30P, A53T, E46K, H50Q, G51D, and A53E. The A53T and A30P mutations affect the response to oxidative stress, with expression of these mutant isoforms significantly increasing cytotoxicity induced by hydrogen peroxide and 1-methyl-4-phenylpyridinium (MPP+) in comparison to cells expressing wild-type α-syn and control cells [33]. Moreover, the A30P, A53T and H50Q mutations result in increased oligomerization and fibril formation compared to wild-type [34,35], conversely, G51D and A53E mutations reduce the aggregation of α-syn [36,37]. E46K mutation disrupts macroautophagy via inactivation of JNK1-Bcl-2 pathway [38]. Recently, strong association was shown between α-syn and sporadic PD in GWAS [39,40]. α-Syn is also a major component of LBs [41]. These arguments illustrate that α-syn is a central player in the pathogenesis of PD.

Although the mechanisms by which the genetic variants affect protein pathology remain to be resolved, the processes by which α-syn protein can become pathological are better understood. Under certain conditions, α-syn monomers interact to form prefibrillar or protofibrils, which in turn can form insoluble fibrils [42,43,44]. A widely accepted hypothesis for α-syn toxicity proposes that protofibrils of α-syn are cytotoxic, whereas the fibrillar aggregates of the protein could represent a cytoprotective mechanism in PD [45]. Supporting this hypothesis, α-syn protofibrils are increased in the brains of patients with PD and dementia with Lewy bodies (DLB) [46], and have been associated with neurotoxicity in α-syn overexpressing cells and mouse models [47,48].

### 2.2. α-Synuclein and Cytotoxicity

**Figure 1:**
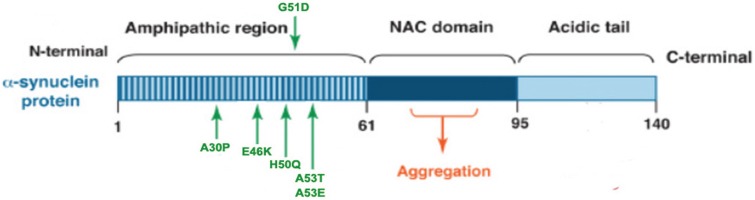
α-Synuclein (α-syn) protein domain structure. α-Syn is a 140 amino acid protein and its sequence can be divided into three regions with distinct structural characteristics. The highly conserved N-terminal domain encodes for a series of imperfect 11 amino acid repeats with a consensus motif of KTKEGV reminiscent of the lipid-binding domain of apolipoproteins, which in certain conditions forms amphipathic helices. The six missense mutations known to cause familial PD (A30P, E46K, H50Q, G51D, A53E and A53T) lie in the amphipathic region, suggesting an important function for this region of the protein. The central hydrophobic region (non-amyloid-β component or NAC domain) of α-synuclein is associated with an increased propensity of the protein to form fibrils [58]. The acidic C-terminal tail contains mostly negatively charged residues and is largely unfolded.

α-Syn is a 14 kDa natively unfolded protein encoded by the *SNCA* gene that is highly conserved in vertebrate species, and the small protein is abundantly expressed in the brain as well as in multiple other central and peripheral tissues [49]. Maroteaux *et al.* [50] first described the localization of α-syn to the nucleus and the presynaptic terminal. Although the exact function of α-syn remains unknown, substantial evidence suggest that α-syn associates with vesicular and membranous structures and plays a role in synaptic vesicle recycling, and storage and compartmentalization of neurotransmitters [51]. The α-syn peptide has 140 amino acids, with three distinct regions: a N-terminal (1–60 residues) that contain four imperfect repeats of KTKEGV motifs; a NAC region (61–95 residues), with three additional KTKEGV repeats; and the hydrophobic and amyloidogenic NAC region, a C-terminus (96–140 residues) that is enriched in acidic and proline residues, and that facilitates interactions with different proteins [52] (see Figure 1). All three regions are necessary for the misfolding of the protein.

The conformation of α-syn is highly dependent on environmental conditions. Indeed, despite the overwhelming evidence that α-syn is a disordered monomer in solution, two recent reports suggest that the native protein exists as a helical tetramer with a molecular weight of about 58 kDa under physiological conditions, with reduced aggregation tendencies, and that the dissociation of the tetramer into monomeric subunits promotes toxic aggregation [53,54]. What is more certain is the α-syn protein adopts oligomeric and/or fibrillar conformations in certain pathological conditions [55,56,57] (such as mutations in the SNCA gene, structural modification produced by environmental changes, mistakes on the post-translational modifications and induction of protein misfolding by seeding or cross-seeding mechanisms).

Up to now, although the precise mechanisms of cytotoxicity of α-syn are still not fully understood, abundant evidence suggests that the toxic effect of α-syn involves many different mechanisms: (i) loss of the normal function of α-syn in neurotransmission release, especially in the regulating DA release [59,60,61,62,63], leads to the selective dysfunction or degeneration of DA neurons in PD; (ii) α-Syn impairs mitochondrial structure and inhibits complex I activity, promoting the production of reactive oxygen species (ROS) [64,65,66,67,68]; (iii) α-Syn disrupts ER-Golgi vesicular transport and results in toxic ER stress [69,70,71]; (iv) α-Syn inhibits protein degradation systems, including ubiquitin-proteasomal system (UPS) and Autophagy-lysosome system [72], interferes with the normal physiology of the cell, and eventually leads to cell injury and death; (v) α-Syn promotes the permeabilization of membranes and disrupts cellular homeostasis [73]; (vi) α-Syn induces the increase of ROS by direct or indirect interaction with oxidative stress [74]; and (vii) “prion-like” propagation, by which pathological α-syn aggregates are propagated between connected brain regions via a cell-to-cell transmission mechanism, which is an important mechanism to induce progressive DA neurons loss in PD [75].

### 2.3. α-Synuclein Post-Translational Modifications

Several post-translational modifications of α-syn may occur, such as phosphorylation, truncation, ubiquitination, nitration. Studies investigating the phosphorylation of α-syn in diseased and aged brains have shown that α-syn can be phosphorylated at serines (S87, S129) as well as at several tyrosines, including Y125, Y133, and Y136. The pS129 modification is most often correlated with PD pathology. This notion is primarily supported by the finding that the majority of α-syn in LBs in postmortem PD brains is phosphorylated at S129 (pS129) [76,77]. The S129 phosphorylation of α-syn in aggregates has also been observed in animal models of PD [78,79]. Although a few studies reported that overexpression of the phosphorylated serine 129 isoform in animal models does not produce toxicity [80,81], most evidence suggests that phosphorylation of α-syn at serine 129 to promote its aggregation and neurotoxicity [82]. Mechanistic studies have shown that aggregated forms of α-syn are more prone to phosphorylation and that pS129 phosphorylated aggregates accumulate as the disease progresses [83,84,85], suggesting that the degree of α-syn pS129 phosphorylation is an indicator of disease progression. The link between S129 phosphorylation and PD pathology has fueled an interest in modulating α-syn phosphorylation at S129 as a potential therapy for PD. Multiple kinases have been identified which phosphorylate α-syn at S129, with most evidence pointing to polo-like kinase 2 (PLK2) as the primary phosphorylated of α-syn S129 [86]. A straightforward therapeutic approach based on reducing α-syn phospho-S129 would be to inhibit PLK 2 kinase activity; however some contradictory findings should be taken into account. For instance, overexpression of PLK 2 in rat brain using adeno-associated viral vectors can suppress α-syn toxicity by promoting autophagy-mediated degradation of phospho-S129 α-syn [87]. Therefore, therapies based on modulating α-syn phospho-S129 appear to require an optimal phosphorylation level rather than a complete dephosphorylation.

The majority of ubiquinated α-syn is mono- to tri-ubiquinated, and while poly-ubiquitination serves as a signal for α-syn degradation by the proteasome, it does not seem to be required for α-syn fibrillation and LB formation [88]. Nevertheless, an interplay between phosphorylation and ubiquitination may render the protein more susceptible to aggregation [89]. Small amounts of various C-terminal truncated forms of α-syn have been detected in LBs, which exhibit greater fibrillation capacity [90]. Oxidation leads to other common post-translational modifications, including nitrosylation, and it has been shown that oxidative stress can stabilize oligomeric α-syn species via the formation of di-tyrosine cross-links [91].

### 2.4. α-Synuclein and Oxidative Stress

Abundant evidence suggests that there is a potential interaction between α-syn and oxidative stress. Some studies have found that oxidative stress induces up-regulation of the expression of α-syn, and promotes its fibrillization and aggregation [92]. Conversely, a high degree of fibrillization and aggregation of α-syn results in an increase of reactive oxygen species (ROS) and neurotoxicity [93]. It is believed that a vicious cycle between α-syn and oxidative stress may play a pivotal role in the progressive loss of SN dopaminergic neurons in PD. For example, incubation of recombinant α-syn with cytochrome c in the presence of H_2_O_2_ [94], or exposure of cells in culture to H_2_O_2_ and ferrous iron, MPP+, NO and superoxide promotes α-syn aggregation [58,95,96]. Additionally, oxidative stress can cause nuclear membrane modifications and α-syn translocation to the nucleus where it can form complexes with histones leading to its oligomerization into insoluble fibrils [97,98,99]. On the other hand, aggregation and high levels of α-syn have been shown to induce oxidant production or increase the level of oxidative stress. As a result, experiments with cultured cells or animal models have demonstrated that over-expression of wild-type or mutant α-syn increases sensitivity to DA, MPTP and 6-OHDA toxicity [100,101,102,103]. Conversely, mice lacking α-syn demonstrate marked resistance to MPTP and other mitochondrial toxins such as malonate and 3-nitropropionic acid [104,105]. Addition of exogenous α-syn fibrils in culture medium or injecting it into the brain can led to selective decreases in synaptic proteins, progressive impairments in neuronal excitability and connectivity, and eventually, neuron death. These results suggest that oxidative stress promotes α-syn aggregation, which in turn increases oxidative stress level, creating a vicious cycle leading to neurodegeneration.

## 3. Parkinson’s Disease and Neuromelanin

### 3.1. Neuromelanin Structure and Biosynthesis

NM is the dark insoluble macromolecule that confers the black (SN) or grey (locus coeruleus) color to monoaminergic basal ganglia. It is a polymer pigment synthesized that contains catecholamine-based compounds such as oxidized DA, DA metabolites as well as proteins and lipids. Histological studies showed that NM granules were located in the neuronal perikaryon and were surrounded by a double membrane [106]. In the SN, NM accumulates during aging and is found after the first two to three years of life [107]. X-ray diffraction studies have shown that NM has a multilayer (graphite-like) three-dimensional structure similar to synthetic and naturally occurring melanin [108]. The three-dimensional structure is derived from planar overlapped sheets consisting of cyclic molecules of indole benzothiazine rings. However, these sheets are stacked much higher in NM than in any other synthetic and naturally occurring melanin [108].

Biosynthesis of NM may due to tyrosine hydroxylase-mediated oxidation of DA [109] or auto-oxidation of DA [110]. Although the process of NM formation is obscure, a recent *in vitro* study has clearly established some steps of this complex process [111]. The study demonstrated that NM synthesis was induced in rat SN neurons and PC12 cell cultures by exposure to L-dopa. The pigment produced in this model contains a stable free radical; in addition, both light and electron microscopy have shown that the pigment synthesized in these cells appears to be identical to human NM, and the granules are surrounded by a double membrane, similar to the naturally occurring NM of the SN. In this model, NM synthesis was shown to be driven by an excess of cytosolic catecholamines.

### 3.2. Neuromelanin Involved in the Pathogenesis of Parkinson’s Disease

Although the role of NM remains unclear, especially in the SN, both under physiological conditions and in the pathogenesis of PD, it is a fact that NM containing neurons in SN are more vulnerable than the non-pigmented ones in PD. Normally, NM is considered to play a protective role intracellular by binding toxic metabolite produced in SN cells such as oxidized DA, DA metabolites and metals [112,113,114] and serve as an antioxidant [115,116]. However, it has been also suggested that NM can be potentially toxic to DA neurons, by directly inhibiting proteasomes function [117], and catalyzing the production of free radicals [118]. NM might also become a source of free radicals by reaction with hydrogen peroxide [119,120]. Changed structure and density of NM have been found in early PD, suggesting that NM may play role in the pathogenesis in PD. Analysis of NM in the SN of PD patients has shown an early accumulation and overload of iron, which can potentially result in increased oxidative stress [121]. The interaction of NM with α-syn has also been suggested as a mechanism for this pigment to modulate neuronal vulnerability [74]. α-Syn is over-expressed in individual melanized neurons [29], and its aggregates redistribute to NM in the SN early in PD but not in healthy controls [121]. Moreover, NM that leaks from degenerating neurons may contribute to the neurodegenerative process by activating microglia [122]. The activated microglia produces proinflammatory cytokines such as TNF-α, IL-6 and NO and may involve in PD pathogenesis [123].

### 3.3. Interaction of Neuromelanin with Organic or Inorganic Molecules

NM interacts with numerous organic and inorganic molecules including lipids, pesticides, toxic compounds and metal ions [124]. NM might reduce the toxicity of MPTP by accumulating its toxic metabolite MPP+ *in vivo* [125]. The herbicide paraquat has a molecular structure similar to that of MPTP, and has been proposed as a Parkinson’s disease inducing agent. The pesticide is accumulated in NM containing nerve cells, where it appears that the NM adsorbed intraneuronal paraquat, protecting the neurons from consequent damage [126]. NM can also accumulate chlorpromazine, haloperidol, and imipramine, thereby contributing to the control of the intraneuronal concentration of these molecules. Because higher intraneuronal concentrations of dopaminergic drugs might be toxic to substantia nigra neurons, NM can influence this toxicity [127]. The association of NM with lipids has been described in several studies [128,129,130]. Although previous studies proposed that lipids were part of the NM molecule, recent work has shown that NM contains about 20% adsorbed lipids. Cholesterol is a minor component in this lipid mixture, with the major component being a new class of polyunsaturated lipid with a high molecular mass, low volatility, and low oxygen content [129]. It may be that NM itself catalyzes the synthesis of this type of lipid. Alternatively, NM could originate from lipofuscin by an enzymatic reaction occurring in lysosomes [131], although this hypothesis is not supported by previous observations [123]. In this case, high molecular mass lipids could be derived from a lysosomal metabolic pathway and might interact with NM within these organelles. The ability of NM to bind different types of organic compounds may influence the intracellular role of lipids and proteins.

High concentrations of iron and other non-alkaline metals are present in several brain nuclei. NM from the SN can interact with many heavy metal ions such as zinc, copper, manganese, chromium, cobalt, mercury, lead, and cadmium; in addition, it binds iron particularly strongly [132,133]. In the course of PD, the concentration of iron in the SN increases by 30%–35%. This accumulation of SN iron seems to occur within the NM granules: the concentration of iron in these granules is higher in patients with PD than in normal subjects [134]. It seems that the amount of iron bound to NM determines whether this molecule acts as a protective agent blocking redox active metal ions or whether in the presence of excess iron it promotes the formation of cytotoxic radicals [135,136]. The ability of NM to chelate other redox active metals such as copper, manganese, chromium, and toxic metals including cadmium, mercury, and lead [133] strengthens the hypothesis that NM may be a high capacity storage trapping system for metal ions and, as such, may prevent neuronal damage. The capacity of NM to form stable complexes with toxic exogenous metals and redox active metals seems to play a protective role even if such a capability can in principle be saturated by high cytosolic concentrations of metals.

## 4. Interaction between α-Synuclein and Neuromelanin

### 4.1. Accumulation of Nenromelanin Increases the Level of α-Synuclein in SN Neurons

NM is considered to play a protective role as a scavenger to clear toxic metabolite produced in SN neurons. However, it has been also suggested that NM can be potentially toxic to DA neurons, by directly inhibiting proteasomal function and catalyzing the production of free radicals [127]. Recently, several studies suggested NM induces the expression or aggregation of α-syn in DA neurons of SN in aging or PD brain [29,115,137,138]. Fasano *et al.* reported that NM directly binds with α-syn in residual SN neurons of PD [139]. The fact that α-syn is expressed in melanoma and nevus, but not in non-melanocytic cutaneous carcinoma and normal skin, suggests that melanin may induce the expression of α-syn [138]. Moreover, α-syn mRNA levels were detected significantly elevated in individual NM-containing DA neurons from PD midbrain when compared to those from matched controls, further supporting the notion of NM induces the expression of α-syn [137]. NM also increases the accumulation of α-syn in DA neurons. Halliday GM *et al.* reported that more vulnerable SN A9 neurons with normal morphological appearance exhibited significantly increased NM density associated with a concentration of α-syn to the lipid component of NM in early PD patients [115]. The increased concentration of neuronal NM and α-syn in normal A9 neurons may already predispose these neurons to precipitate α-syn around NM-associated lipid under oxidative conditions. These changes may trigger a cascade of events leading to larger intracellular aggregates of α-syn and eventually lead to the death of those NM-containing DA neurons in PD [115]. In addition to PD brain, our previous study found that NM accumulation also induces expression of α-syn during aging in NM-containing DA neurons and leads to age related loss of DA neurons [29]. In our previous study, the marked increase in NM and α-syn in aged individuals suggests that NM content may be essential for α-syn over-expression [29]. We found that accumulation of NM in SN starts very early in life [140], whereas the detectable pathogenic accumulation of α-syn begins around middle age [141] in the same area. Although both regions contain DA neurons and are closely localized to one another, it has been reported that α-syn accumulates with age in the SN but not in the ventral tegmental area (VTA) [141]. An important difference between the VTA and the SN is that the dopaminergic neurons in the SN contain significantly more NM than those in the VTA. This further supports the notion that NM may be responsible for age-associated increases in α-syn.

Although growing evidence suggests that excessive accumulation of NM may initiate gathering of α-syn, the precise mechanism is still unclear. Findings to date suggested that iron saturated NM produces many free radical species and increases oxidative insults [142], which may initiate gathering of α-syn. Alternatively, accumulated NM could induce proteasome inhibition [111,143] and result in a reduction of α-syn clearance. Further, NM may be synergistic with other factors that promote age-related “autophagic stress” [144] and thereby influence the degradation of α-syn [145].

### 4.2. α-Synuclein Induces the Biosynthesis of Neuromelanin

Pan *et al.* [146] reported that over-expressed α-syn increased the content of NM in SH-SY5Y and PC12 dopaminergic cells, suggesting α-syn may promote the biosynthesis of NM in DA neurons. However, the precise mechanisms of α-syn induce biosynthesis of NM is still unclear. At present, it has been accepted that the formation of NM is due to either enzymatically mediated or auto-oxidation of DA pathway [147]. So far, tyrosinase, tyrosine hydroxylase (TH), peroxidase, prostaglandin H synthase, and macrophage migration inhibitory factor were suggested to be involved in NM synthesis [148]. However, there is currently no general agreement on the role eventually played by any of these enzymes in the synthesis of NM in SN neurons [147]. Although the role of these enzymes in the synthesis of NM is not known for sure, current studies indicate that α-syn modulates the activities of some of them, such as tyrosinase [149], tyrosine hydroxylase (TH) [150], and peroxidase [151]. α-Syn may affect the biosynthesis of NM by regulating these enzyme activities.

On the other hand, NM could derive from non-enzymatic oxidation. The auto-oxidation of catechols to quinones with the subsequent addition of thiol groups has been demonstrated in the brain [152]. An *in vitro* study clarified some steps of the complex biosynthesis of NM in cultured dopaminergic neuronal cells [123]. NM synthesis could be induced in rat SN and in PC12 cells in culture by exposure to L-DOPA, which is rapidly converted in DA in the cytosol. The pigment produced in this model was chemically identical to human NM as demonstrated by EPR. Moreover, it was localized in characteristic organelles surrounded by a double membrane, as is similar to naturally occurring NM [123]. Moreover, NM synthesis could be inhibited by the adenoviral-mediated over-expression of the synaptic vesicular monoamine transporter 2 (VMAT2), which sequesters monoamines from the cytosol into synaptic vesicles for subsequent neurotransmission [153], suggesting that an excess of cytosolic free catecholamines is an essential factor for the synthesis of NM. The high concentration of NM in SN neurons seems to be linked to the presence of considerable amounts of cytosolic dopamine that have not been sequestered into synaptic vesicles [123].

Abundant evidence has proved that α-syn is involved in regulating DA homeostasis and affecting the levels of cytosolic DA. α-Syn may modulate DA homeostasis through multiple mechanisms, such as synthesis, storage, release and reuptake [101]. α-Syn regulates the biosynthesis of DA by inhibiting the activity of tyrosine hydroxylase (TH) [154], the rate-limiting enzyme responsible for converting tyrosine to L-3,4-dihydroxyphenylalanine (L-DOPA) in the DA synthesis pathway. Overexpression of α-syn has also been reported to decrease the rate of DA release, both in mouse and cell culture models [155,156]. α-Syn can direct bind to DAT via its C-terminal domain and enhance extracellular DA uptake by increasing the number of functional transporters at the cell surface [157,158]. Enhanced extracellular DA uptake increases the levels of cytosolic DA. More importantly, α-syn may affect the storage of DA by regulating vesicle docking and recycling at the nerve terminal, increasing permeability of secretory vesicles and decreasing the levels of VMAT2. This could prevent incorporation of newly synthesized and newly taken up DA into vesicles and lead to increase the level of cytosolic DA [159]. The notion is supported by some studies that found α-syn mutation or overexpression lead to elevated cytosolic DA [160,161]. The cytosolic DA can be metabolized in mitochondria by monoamine oxidase (MAO). If these processes are insufficient to control cytosolic DA homeostasis, excess cytoslic DA can be oxidized to quinones and semiquinones via iron catalysis in the cytosol. These quinones react with Cys residues in proteins, such as α-syn [162], to form DA-Cys-protein adducts, which are phagocytized in autophagic vacuoles (AG). Because of a low level of lysosomal fusion and/or to a buildup of undegradable DA-adducts, even in the presence of lysosomal hydrolases, over time the vacuoles become NM granules [148]. So, α-syn may induce the biosynthesis of NM by increasing the levels of cytosolic DA.

## 5. Conclusions

The pathological characteristics of PD are progressive loss of neuromelanin (NM)-containing dopaminergic neurons in SNpc and α-syn positive LBs in survival neurons. Abundant evidence has proved that α-syn play a pivotal role in the pathogenesis of PD. Missense mutations in α-syn gene could result in early onset familial PD. The toxicity of abnormal α-syn forms to DA neurons and loss of normal functions of this protein are important causes in the pathogenesis in sporadic PD. NM is a dark, complex, and insoluble pigment granule in several types of neurons of the central nervous system that is particularly concentrated in the DA neurons of SN and in the noradrenergic neurons of locus coeruleus [127]. Although the mechanism is not clear, SN and the locus coeruleus are the two brain areas mostly affected by PD. This fact draws a close attention to NM and its possible involvement in neurodegenerative processes. Recently, several studies suggested that the interaction of NM with α-syn may be a mechanism for this pigment to modulate neuronal vulnerability. α-Syn is over-expressed in individual melanized neurons [29,137,138] and its aggregates redistribute to NM in the SN early in PD but not in healthy controls [115]. On the other hand, α-syn also induces the biosynthesis of NM by increasing the levels of cytosolic DA [146].

**Figure 2:**
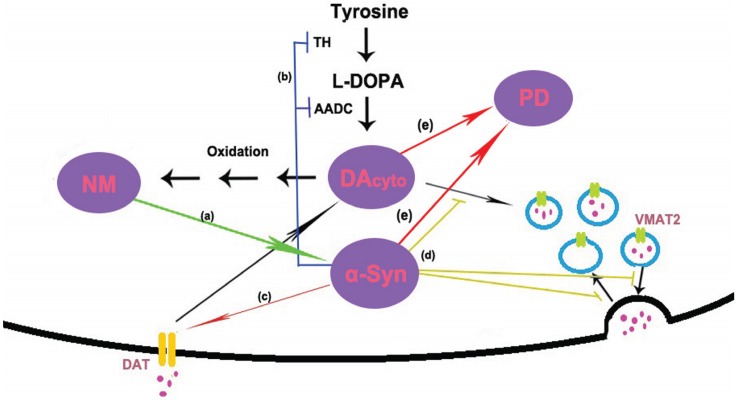
Model of possible interactions between α-synuclein (α-syn) and neuromelanin (NM). (**a**) NM induces the expression and aggregation of α-syn; (**b**) dopamine (DA) is synthesized in the cytoplasm by the action of tyrosine hydroxylase (TH) and amino acid decarboxylase (AADC). α-Syn has been shown to regulate the activity of TH and AADC; (**c**) DA is reuptake via the dopamine transporter (DAT). Studies in cell culture systems have shown that α-syn is necessary for the trafficking of DAT to the cell surface. (**d**) Once synthesized, DA is immediately sequestered into vesicles by the vesicular monoamine transporter 2 (VMAT2). Several lines of evidence suggest that α-syn is involved in regulating storage, release and presynaptic vesicle cycling. (**e**) High levels of α-syn and cytosolic DA cause selective DA neuron death and eventually lead to PD.

In conclusion, no matter what is the trigger, NM and α-syn are likely to form a vicious cycle of mutual promotion, eventually the untoward cycle results in the death of DA neurons in PD (see Figure 2). In sporadic PD, age-related accumulation of NM induces α-syn expression and aggregation. On the other hand, accumulated α-syn may promote the biosynthesis of NM by increasing the levels of cytosolic DA. In familial PD, the mutants of α-syn also can trigger the accumulation of NM and start this vicious cycle.
